# Corrigendum: Human abdominal subcutaneous-derived active beige adipocytes carrying *FTO* rs1421085 obesity-risk alleles exert lower thermogenic capacity

**DOI:** 10.3389/fcell.2023.1249909

**Published:** 2023-08-30

**Authors:** Attila Vámos, Rini Arianti, Boglárka Ágnes Vinnai, Rahaf Alrifai, Abhirup Shaw, Szilárd Póliska, Andrea Guba, Éva Csősz, István Csomós, Gábor Mocsár, Cecilia Lányi, Zoltán Balajthy, László Fésüs, Endre Kristóf

**Affiliations:** ^1^ Laboratory of Cell Biochemistry, Department of Biochemistry and Molecular Biology, Faculty of Medicine, University of Debrecen, Debrecen, Hungary; ^2^ Doctoral School of Molecular Cell and Immune Biology, University of Debrecen, Debrecen, Hungary; ^3^ Universitas Muhammadiyah Bangka Belitung, Pangkalanbaru, Indonesia; ^4^Genomic Medicine and Bioinformatics Core Facility, Department of Biochemistry and Molecular Biology, Faculty of Medicine, University of Debrecen, Debrecen, Hungary; ^5^Proteomics Core Facility, Department of Biochemistry and Molecular Biology, Faculty of Medicine, University of Debrecen, Debrecen, Hungary; ^6^Department of Biophysics and Cell Biology, Faculty of Medicine, University of Debrecen, Debrecen, Hungary; ^7^ Laser Clinic, Budapest, Hungary

**Keywords:** adipocytes, beige, obesity, FTO rs1421085, thermogenesis, UCP 1, *SLC7A10*

In the published article, there was an error. In the published article, the **Reference** “Bjune et al., 2005” was cited with an incorrect year of publication. The correct year of publication is 2019.

In the published article “Bjune, J. I., Haugen, C., Gudbrandsen, O., Nordbø, O. P., Nielsen, H. J., Våge, V., et al. (2019). IRX5 regulates adipocyte amyloid precursor protein and mitochondrial respiration in obesity. *Int J Obes (Lond).*, 43(11), 2151–2162. https://doi.org/10.1038/s41366-018-0275-y” was not referenced in the article. The reference has now been inserted into the article.

A correction has been made to the **Introduction**. This sentence previously stated:

“In addition, *IRX5* silencing increased the mitochondrial respiration in isolated mouse adipocytes (**Bjune et al., 2005**).”

The corrected sentence appears below:

“In addition, *IRX5* silencing increased the mitochondrial respiration in isolated mouse adipocytes (Bjune et al., 2019).”

The authors apologize for this error and state that this does not change the scientific conclusions of the article in any way. The original article has been updated.
